# Does the stress response predict the ability of wild birds to adjust to short-term captivity? A study of the rock pigeon (*Columbia livia*)

**DOI:** 10.1098/rsos.160840

**Published:** 2016-12-21

**Authors:** Frédéric Angelier, Charline Parenteau, Colette Trouvé, Nicole Angelier

**Affiliations:** Centre d'Etudes Biologiques de Chizé, CNRS-ULR, UMR-7372, 79360 Villiers-en-Bois, France

**Keywords:** corticosterone, stress, captivity

## Abstract

Although the transfer of wild animals to captivity is crucial for conservation purposes, this process is often challenging because some species or individuals do not adjust well to captive conditions. Chronic stress has been identified as a major concern for animals held on long-term captivity. Surprisingly, the first hours or days of captivity have been relatively overlooked. However, they are certainly very stressful, because individuals are being transferred to a totally novel and confined environment. To ensure the success of conservation programmes, it appears crucial to better understand the proximate causes of interspecific and interindividual variability in the sensitivity to these first hours of captivity. In that respect, the study of stress hormones is relevant, because the hormonal stress response may help to assess whether specific individuals or species adjust, or not, to such captive conditions (‘the stress response-adjustment to captivity hypothesis’). We tested this hypothesis in rock pigeons by measuring their corticosterone stress response and their ability to adjust to short-term captivity (body mass loss and circulating corticosterone levels after a day of captivity). We showed that an increased corticosterone stress response is associated with a lower ability to adjust to short-term captivity (i.e. higher body mass loss and circulating corticosterone levels). Our study suggests, therefore, that a low physiological sensitivity to stress may be beneficial for adjusting to captivity. Future studies should now explore whether the stress response can be useful to predict the ability of individuals from different populations or species to not only adjust to short-term but also long-term captivity.

## 1. Introduction

In the context of biodiversity loss, the ability to bring wild animals into captivity has growing conservation implications [1,2]. For example, several conservation programmes bring some wild animals into captivity for breeding to facilitate population growth for endangered populations. Wild animals are also often kept in captivity in zoological parks for recreational and educational purposes. Similarly, animals injured or exposed to environmental pollutants are regularly treated in rehabilitation centres before their release into their natural environment. Importantly, some species and individuals do not seem to adjust as well as others to these artificial conditions and several wild animals suffer from stress when held in captivity [2]. Such stress jeopardizes the success of these programmes [3] because it is associated with reproductive failure, pathologies and even death of captive animals [4].

There is much evidence that chronic stress is one of the main causes of the failures of many reintroduction, translocation or captive conservation projects [2,5]. Surprisingly, the influence of the initial acute stress of being transferred to captivity has been less studied relative to chronic stress and long-term captivity. However, the first hours or days of captivity are probably crucial to consider, because individuals are confronted with a totally novel confined environment that is certainly stressful. Thus, body mass loss is especially pronounced during the first days of captivity [6], and, as a result of acute stress or/and energetic stress, mortality may even occur at capture or in the first hours/days of captivity [2,7].

In that respect, it appears crucial to assess this sensitivity to captivity before animals are brought into captive conditions [2]. Studying the hypothalamic–pituitary–adrenal axis (HPA) appears promising because it certainly plays a major role in the ability of individuals to adjust to stress and captivity [8–12]. In response to an acute stressor, the activation of this axis results in increased synthesis of glucocorticosteroids (GCs) that mediate behavioural and physiological changes to help the organism cope with the stressor [13]. Despite immediate benefits, this stress response can also entail important costs, because elevated GC levels impair behavioural and physiological functions when maintained during a prolonged period [4,14–16]. In the 1990s, an elegant protocol was proposed to study this hormonal stress response [17]. It consists of capturing an individual, taking an initial blood sample to monitor baseline GC levels, and then restraining it for a short amount of time to mimic a stressor. At the end of this restraint, a final blood sample is collected to monitor the increase in circulating GC levels that results from this standardized stress protocol [17]. Importantly, this increase in GC levels is thought to be a reliable proxy of the sensitivity of an organism to stressors [16], and therefore, it could be useful to determine whether a low physiological sensitivity to stress is necessary for wild animals to adjust to captivity (‘the stress response-adjustment to captivity hypothesis’; see also [11]).

If this hypothesis is supported, it could provide important benefits to managers and scientists to predict which species or individuals are likely to cope well with a transitory period of captivity. To the best of our knowledge, this hypothesis has, however, never been tested so far. For obvious ethical reasons, it appears crucial to first test this hypothesis with a relevant common wild bird species. We thus tested this hypothesis in rock pigeons (*Columbia livia*) by measuring their corticosterone response to a standardized stress protocol [17], and then by subsequently transferring them into captivity for a day. Rock pigeons are especially relevant to test this hypothesis for several reasons. First, a preliminary study showed that rock pigeons are affected by the stress of being transferred to captivity (Angelier *et al*. 2014, unpublished data). Second, there is a large interindividual variation in the corticosterone stress response in this species [18], allowing us to test whether this physiological measure is related to adjustment to short-term captivity. Finally, this species is not endangered, does not show a high risk of mortality when transferred to captivity and is therefore ethically relevant to test this hypothesis for the first time. According to ‘the stress response-adjustment to captivity’ hypothesis, we predicted that individuals will not tolerate captive conditions well if they respond strongly to the standardized stress protocol (i.e. an important increase in corticosterone levels). First, we measured the change in body mass following one day in captivity. Mass loss is considered as one of the most reliable proxies of the inability of vertebrates to adjust to captivity [3,6,11,19,20]. We therefore predicted that the corticosterone response to a stress protocol will be positively correlated to the body mass loss that the pigeons experienced after a day of captivity. Second, we measured circulating corticosterone levels of pigeons after a day of captivity. Because elevated corticosterone levels are usually an indicator of stress ([4,11], but see also [3,20]), we predicted that the corticosterone response to a stress protocol would be positively correlated with corticosterone levels of pigeons after a day of captivity.

## 2. Material and methods

Fieldwork was carried out in 2014 in a moderately urbanized area (43°34′N, 7°02′E). Twenty-nine pigeons were captured using Potter traps (13 females and 16 males) [18] and immediately bled according to a standardized stress protocol [17]: an initial blood sample (0.3 ml) was collected within 3 min. A second sample was taken 60 min after the first (0.3 ml) when stress-induced corticosterone levels have reached their maximum [17]. Pigeons were kept in cloth bags between the initial and second blood samples to mimic an acute stress [17]. All birds were weighed with a scale (±1 g). Their skull and tarsus lengths were measured with a calliper (±0.1 mm).

Pigeons were then transferred to an aviary where they were provided with food and water ad libitum. A day after their capture, they were captured and immediately bled (within 3 min, 0.3 ml) to assess corticosterone levels after a day of captivity. They were then held in individual cloth bags for 60 min and were bled again (0.3 ml) to measure their corticosterone response to a standardized stress protocol after a day of captivity. This second stress protocol allowed us to check that the HPA axis was still able to secrete more corticosterone if an additional stressor occurred. All pigeons were weighed to measure body mass change following a day of captivity. To further test the dynamic of body mass change of captive birds, we brought seven pigeons in captivity in 2016 and measured their body mass every day during a week (electronic supplementary material, S1). No bird died during these experiments, and all birds were released after these experiments and were seen at the field site during the following days or weeks.

Blood samples were centrifuged, and plasma was stored at −20°C until assayed. Plasma concentrations of corticosterone were determined by radioimmunoassay, as previously described [21]. Briefly, plasma corticosterone was measured in samples after ethyl ether extraction by radioimmunoassay. Duplicate aliquots of the extracts were incubated overnight at 4°C with ^3^H-corticosterone and antiserum. The bound and free corticosterone fractions were separated by adding dextran-coated charcoal. After centrifugation, the bound fraction was counted in a liquid scintillation counter. All samples were run in one assay (intra-assay variation: 8.31%).

Statistical analyses were conducted with SAS software (v. 9.4). First, we tested the influence of the time of sampling (at capture, after the first stress protocol, after a day of captivity and after the second stress protocol), sex and their interaction on corticosterone levels by using mixed models (random factor: bird identity). Contrasts (*t*-tests) were used to compare corticosterone levels through the sampling protocol. Second, we used a generalized-linear model to test whether body mass loss and circulating corticosterone levels after a day of captivity were affected by sex, the initial individual stress response (i.e. stress-induced corticosterone levels reached after the first standardized stress protocol) and their interaction. Baseline and stress-induced corticosterone levels were not correlated with body condition at capture (ANCOVAs, all *p* > 0.05).

## 3. Results

**Figure 1.**
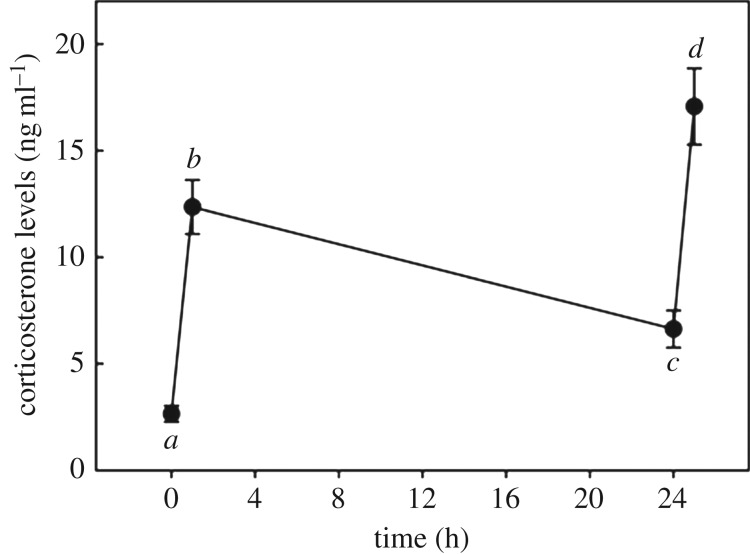
After capture (time = 0 h), corticosterone levels of birds increased in response to a stress protocol and reached high stress-induced corticosterone levels (time = 1 h). Circulating corticosterone levels then decrease while the birds are kept captive, but they did not return to pre-capture baseline corticosterone levels after a day of captivity (time = 24 h). Corticosterone levels finally increased in response to a second stress protocol and reached high stress-induced corticosterone levels (time = 25 h). Different letters denote statistical differences between times of sampling. Data are expressed as means ± s.e.

Circulating corticosterone levels of pigeons were significantly affected by the standardized stress protocol (*F*_3,81_ = 45.12, *p* < 0.001; figure 1). However, they were not affected by sex (*F*_1,81_ = 0.05, *p* = 0.826) and the ‘sex × time of sampling’ interaction (*F*_3,81_ = 1.37, *p* = 0.258). At capture, corticosterone levels significantly increased in response to the standardized stress protocol (*t* = −7.22, *p* < 0.001). After a day of captivity, corticosterone levels significantly decreased from stress-induced levels the day before (*t* = −2.37, *p* < 0.001) but did not return to baseline corticosterone levels (*t* = −2.85, *p* = 0.006). After a day of captivity, corticosterone levels still increased in response to the standardized stress protocol (*t* = −7.92, *p* < 0.001) and reached higher stress-induced levels when compared with the corticosterone levels reached after the first standardized stress protocol (*t* = −3.56, *p* < 0.001).

**Figure 2.**
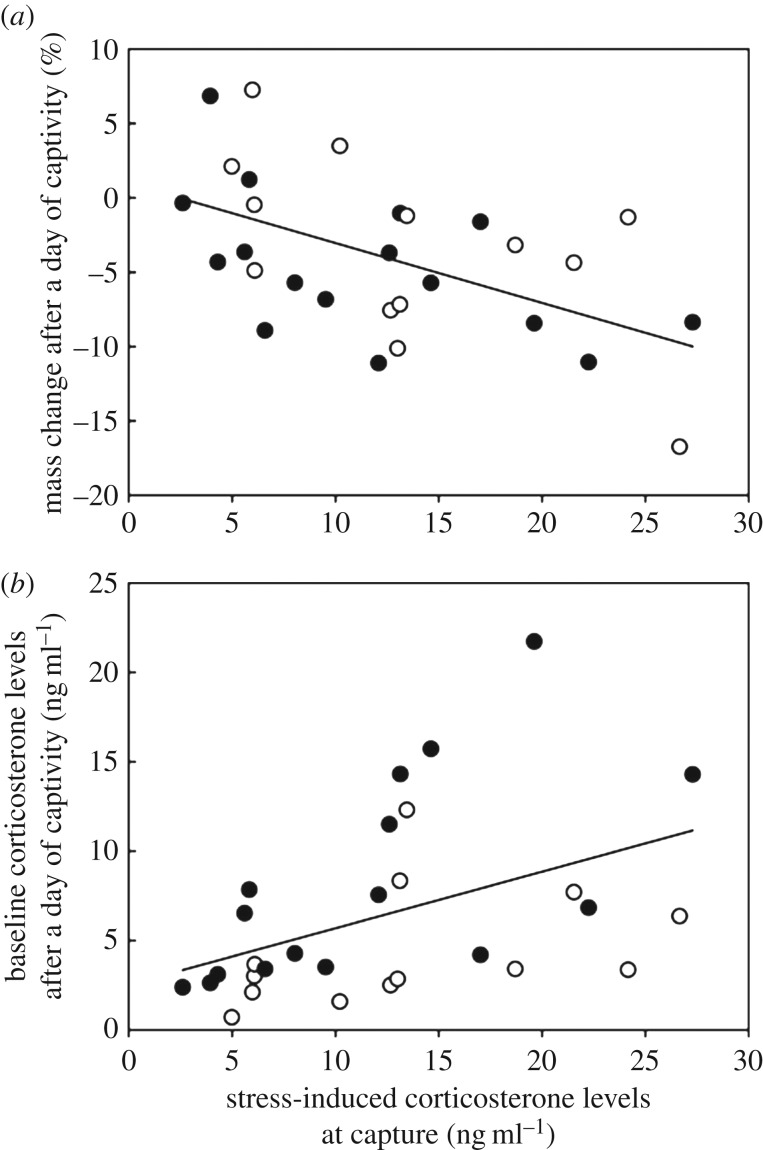
Stress-induced corticosterone levels are positively correlated with (*a*) body mass loss and (*b*) circulating corticosterone levels of pigeons after a day of captivity. Open and filled symbols, respectively, denote females and males.

When transferred to captivity, most birds lost mass within a day (*F*_1,29_ = 16.08, *p* < 0.001). This body mass loss did not differ between sexes (*F*_1,25_ = 1.01, *p* = 0.325). However, it was significantly and positively correlated with the stress-induced corticosterone levels that were measured in response to the standardized stress protocol following capture (*F*_1,25_ = 11.47, *p* = 0.002; figure 2*a*). There was no effect of the ‘sex × stress-induced corticosterone levels' interaction on body mass loss (*F*_1,25_ = 0.28, *p* = 0.603). After a day of captivity, baseline corticosterone levels did not differ between sexes (*F*_1,25_ < 0.01, *p* = 0.973). However, they were significantly and positively correlated with the stress-induced corticosterone levels that were measured in response to the standardized stress protocol following capture (*F*_1,25_ < 10.10, *p* = 0.004, figure 2*b*). There was no effect of the ‘sex × stress-induced corticosterone levels’ interaction on these corticosterone levels (*F*_1,25_ = 2.43, *p* = 0.131).

When pigeons were transferred into captivity for a week, their body mass dropped during the first 2 days of captivity and gradually returned to normal within a week (electronic supplementary material, S1).

## 4. Discussion

We found that the corticosterone response to a standardized stress protocol can be a reliable predictor of the ability of wild individuals to adjust to short-term captivity, supporting therefore ‘the stress response-adjustment to captivity hypothesis’. Thus, this corticosterone stress response was closely associated not only with the ability of pigeons to maintain their body mass when transferred to captivity, but also with the circulating corticosterone levels they exhibit after a day of captivity. Body mass change is considered as one of the most reliable proxies of the ability of individuals to adjust to captive conditions [10,11,19,20]: when transferred to captivity, wild animals often suffer from stress [2,10,20] and, as a result of stress or/and elevated corticosterone levels, they may reduce their food consumption and concomitantly increase their energy expenditure [4,14]. Although we did not measure food consumption or energy expenditure in our study, we found that most pigeons lost mass when transferred to captivity. Importantly, body mass dropped during the first two days of captivity, and then, gradually returned to normal, emphasizing therefore that the first 2 days of captivity are certainly the most constraining, at least for this species. This emphasizes the idea that the hours following transfer to captivity are crucial to consider in future studies. Supporting further the idea that pigeons suffered from stress when transferred to captive conditions, we also found that pigeons had elevated corticosterone levels after a day of captivity despite having water and food ad libitum.

Interestingly, there was a large interindividual variation in these two proxies of stress. First, some pigeons maintained their body mass after a day of captivity, while others showed an increased reduction of their body mass (more than 10%, figure 2). Second, some individuals had elevated corticosterone levels after a day of captivity, whereas others almost returned to pre-captive baseline corticosterone levels (figure 2). Although prolonged stress usually induces an increased secretion of corticosterone in the bloodstream, it is instead sometimes associated with an alteration of the HPA axis and with lowered corticosterone levels [19,20]. However, pigeons still had a functional HPA axis in our study because they were able to mount a corticosterone stress response after a day of captivity. It is possible that elevated corticosterone levels in captive pigeons resulted from a remaining effect of the acute stress protocol that was conducted at capture rather than from the stress of being captive [22]. Thus, captive pigeons may not have had enough time to recover from the restraint stress, explaining potentially why their corticosterone levels did not return to baseline after a day in captivity. Although this hypothesis would need to be specifically tested, it is unlikely to explain our results because several pigeons had low corticosterone after a day of captivity in this study (similar to pre-captive baseline corticosterone levels). Moreover, the corticosterone stress recovery seems to be quite rapid in wild birds [23].

These results suggest that some individuals cope better (or at least faster) with short-term captivity and stressors than others [16]. Importantly, this ability to adjust to short-term captivity is related to the corticosterone stress response that was measured immediately before the transfer of individuals to captivity. Specifically, pigeons with a stronger corticosterone stress response lost more mass when transferred to captivity and had higher circulating corticosterone levels after a day of captivity than those with a weaker stress response. Therefore, the corticosterone response to a stress protocol can be a relevant tool to evaluate the ability of wild individuals to cope with short-term captivity.

The ability of individuals to adjust to short-term captivity has wide implications, because transfer to captivity is a prerequisite of multiple conservation programmes and mortality may occur within the first hours following the transfer of wild animals to captivity [2]. In that respect, our findings support the idea that a low physiological sensitivity to stress may be necessary to adjust to captivity, at least during the first day following the transfer to captivity. Although our study supports ‘the stress response-adjustment to captivity hypothesis’, it only focused on the day following the transfer to captivity while neglecting the importance of long-term captivity. Moreover, it focused on one common species that has gone through several domestication events and that is known to cope pretty well with captivity [24]. Our results suggest that the corticosterone stress response is a promising physiological tool to predict the ability of wild birds to cope with short-term captivity. Further tests of this hypothesis are now needed in other species, including species that are more difficult to hold in captivity. In order to further test this hypothesis, future studies should also concomitantly examine complementary components of the HPA axis (corticosterone, corticosterone-binding globulin, etc. [14,15]) and the ability of wild animals to adjust to short-term but also long-term captivity (through body mass change and behavioural proxies [2,10,11,20]).

Because corticosterone laboratory analyses require some time, it may currently be difficult to measure the corticosterone stress response of individuals quickly enough after the initial capture to determine whether they should be transferred to captivity or not. However, corticosterone laboratory analyses can still be conducted within three hours at the laboratory, allowing managers to take a fairly rapid decision (i.e. deciding to release the bird or to keep it in captivity). Moreover, recent biomedical developments suggest that such assays could be conducted in the field within 10 min in the future [25], opening up exciting prospects regarding the use of corticosterone for conservation programmes. Finally, the corticosterone stress response has already been shown to be affected by numerous factors (e.g. phylogeny, food abundance, body condition, climate, age, parasite load, etc. [16,17]) and, combined with further support for ‘the stress response-adjustment to captivity hypothesis’, these results may provide a rule of thumb to select the species, populations or individuals that can or cannot be transferred to captivity.

## Supplementary Material

ESM1 from "Does the stress response predict the ability of wild birds to adjust to short-term captivity? A study in the rock pigeon (Columbia livia)" by Frédéric Angelier, Charline Parenteau, Colette Trouvé, Nicole Angelier. This table present the data that were used for this article

## Supplementary Material

ESM2 from "Does the stress response predict the ability of wild birds to adjust to short-term captivity? A study in the rock pigeon (Columbia livia)" by Frédéric Angelier, Charline Parenteau, Colette Trouvé, Nicole Angelier. This analysis and thisfigure present the influence of a week of captivity on daily body mass of rock pigeons.

## Data accessibility

Raw data are provided (electronic supplementary material, S2).
