# Supplementary material for: Does the stress response predict the ability of wild birds to adjust to short-term captivity? A study of the rock pigeon (Columbia livia)
Source: R Soc Open Sci. 2016 Dec 21;3(12):160840. doi: 10.1098/rsos.160840 (PMC5210699; doi:10.1098/rsos.160840)
Supplement: ESM1 from "Does the stress response predict the ability of wild birds to adjust to short-term captivity? A study in the rock pigeon (Columbia livia)" by Frédéric Angelier, Charline Parenteau, Colette Trouvé, Nicole Angelier. This table present the data that were used for this article [file rsos160840supp1.pdf]

Sex	CORTA (ng/ml)	CORTB (ng/ml)	CORTACaptivity (ng/ml)	CORTBCaptivity (ng/ml)
M	4.49	5.83	7.84	17.92
M	3.99	13.13	14.31	15.47
F	2.17	6.07	3.00	21.06
M	3.17	27.28	14.28	15.24
M	0.95	4.30	3.10	4.93
F	0.73	24.16	3.36	11.59
M	3.40	9.52	3.51	16.50
F	0.43	18.70	3.40	26.09
M	1.44	12.08	7.55	20.50
M	3.71	19.62	21.72	47.14
M	0.53	3.94	2.62	7.25
M	1.57	22.25	6.84	24.73
F	2.61	21.54	7.70	15.22
M	1.82	12.60	11.50	28.76
F	1.34	10.21	1.58	6.11
F	0.75	12.67	2.50	14.37
F	3.02	6.09	3.67	4.73
M	6.61	14.62	15.72	13.29
M	0.63	6.59	3.40	22.40
F	4.77	13.11	8.34	14.11
M	1.01	2.61	2.38	7.62
M	1.77	17.02	4.19	12.34
F	2.86	26.65	6.36	39.51
F	4.44	13.45	12.30	23.69
M	0.97	8.03	4.28	8.18
F	0.80	5.98	2.11	15.04
F	1.14	13.00	2.85	29.96
M	1.03	5.60	6.52	17.99
F	1.17	4.98	0.70	11.05

Mass change (%)
1.2
-1.0
-0.5
-8.4
-4.3
-1.3
-6.8
-3.2
-11.1
-8.4
6.8
-11.0
-4.4
-3.7
3.5
-7.6
-4.9
-5.7
-8.9
-7.2
-0.4
-1.6
-16.7
-1.2
-5.7
7.3
-10.1
-3.6
2.1
