# Supplementary material for: Does the stress response predict the ability of wild birds to adjust to short-term captivity? A study of the rock pigeon (Columbia livia)
Source: R Soc Open Sci. 2016 Dec 21;3(12):160840. doi: 10.1098/rsos.160840 (PMC5210699; doi:10.1098/rsos.160840)

Angelier F, Parenteau C, Trouvé C, Angelier N 2016 Does the stress response predict the ability of wild birds to adjust to short-term captivity? A study in the rock pigeon (*Columbia livia*). *Royal Society Open Science*

Electronic Supplementary file

Body mass change of wild rock pigeons following transfer to captivity

In August 2016, another experiment was conducted to monitor the dynamic of body mass change following transfer to captivity. The goal was to test how wild rock pigeons react to the stress of captivity through time. This experiment was conducted in a moderately urbanized area (43°34'N, 7°02'E) and seven pigeons were captured using Potter traps and weighed with a scale (±1 g). These pigeons were then transferred to an aviary where they were provided with food and water *ad libitum*. They were kept during 7 days in captivity and their body mass was measured every day to monitor body mass change throughout this period (percent of body mass change relative to the body mass at capture). We tested the influence of the day of measurement on body mass change by using a repeated-measures model.

We found that the body mass of pigeons significantly varied throughout the study period ($F_{5,30}$=7.87, p<0.001). Specifically, it dropped during the first two days of captivity (day 1 and day 2; Fig. 1). Then, it progressively increased and it returned to a body mass (day 6) that was only slightly lower than the body mass that was measured at capture ($F_{1,6}$=6.59, p=0.043; Fig. 1).

These results suggest that the first two days of captivity are the most constraining ones for wild pigeons. In this additional study, pigeons showed a large body mass loss during that period. In contrast, they progressively restore their body mass after these two days, suggesting that they adjust to captivity. Although long-term adjustment to captivity is crucial to study, these additional results highlight the constraints that occur during the hours following transfer to captivity. Therefore, it highlights the importance of studying also the short-term adjustment of wild animals to captivity (i.e. the objective of our main study).

Figure1: Body mass change (%) of rock pigeons following transfer to captivity. Negative values refer to mass loss. Data are expressed as means ± standard errors.

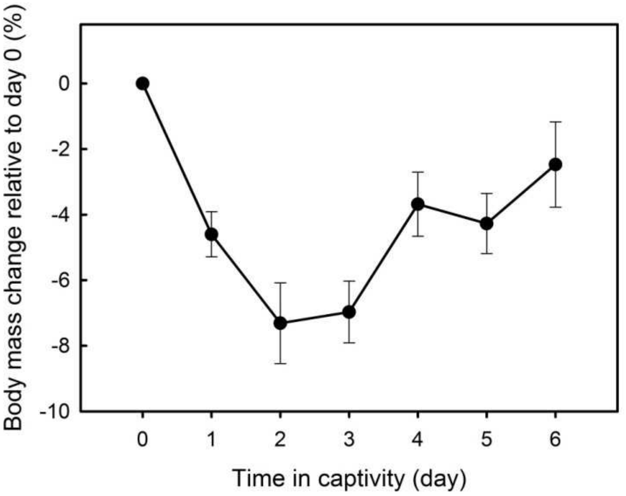

Supplement: ESM2 from "Does the stress response predict the ability of wild birds to adjust to short-term captivity? A study in the rock pigeon (Columbia livia)" by Frédéric Angelier, Charline Parenteau, Colette Trouvé, Nicole Angelier. This analysis and thisfigure present the influence of a week of captivity o [file rsos160840supp2.pdf]
